# Gene Expression Analysis of Zebrafish Melanocytes, Iridophores, and Retinal Pigmented Epithelium Reveals Indicators of Biological Function and Developmental Origin

**DOI:** 10.1371/journal.pone.0067801

**Published:** 2013-07-09

**Authors:** Charles W. Higdon, Robi D. Mitra, Stephen L. Johnson

**Affiliations:** Department of Genetics, Washington University, St. Louis, Missouri, United States of America; Purdue University, United States of America

## Abstract

In order to facilitate understanding of pigment cell biology, we developed a method to concomitantly purify melanocytes, iridophores, and retinal pigmented epithelium from zebrafish, and analyzed their transcriptomes. Comparing expression data from these cell types and whole embryos allowed us to reveal gene expression co-enrichment in melanocytes and retinal pigmented epithelium, as well as in melanocytes and iridophores. We found 214 genes co-enriched in melanocytes and retinal pigmented epithelium, indicating the shared functions of melanin-producing cells. We found 62 genes significantly co-enriched in melanocytes and iridophores, illustrative of their shared developmental origins from the neural crest. This is also the first analysis of the iridophore transcriptome. Gene expression analysis for iridophores revealed extensive enrichment of specific enzymes to coordinate production of their guanine-based reflective pigment. We speculate the coordinated upregulation of specific enzymes from several metabolic pathways recycles the rate-limiting substrate for purine synthesis, phosphoribosyl pyrophosphate, thus constituting a guanine cycle. The purification procedure and expression analysis described here, along with the accompanying transcriptome-wide expression data, provide the first mRNA sequencing data for multiple purified zebrafish pigment cell types, and will be a useful resource for further studies of pigment cell biology.

## Introduction

Pigment cells serve as useful models for understanding many aspects of developmental and cell biology. For example, melanocytes are pigment cells studied to understand cell specification, migration, differentiation, survival, regeneration, organelle transport, secretion, and disease [1]–[15]. Melanocytes produce melanin, which in humans serves as a UV protectant in skin [16]. Melanocytes also have roles in other organs such as the ear, brain, heart, and adipose tissue [17], [18]. The incidence of melanomas, a disease of melanocytes and the most lethal form of skin cancer, is also increasing [19]. Methods to isolate and culture melanocytes for in vitro studies have been informative for understanding melanocyte biology [20], [21]. *In vivo* studies of melanocyte biology and melanoma dynamics have been aided by the identification of mutants in mice and zebrafish[22]–[25]. Given the utility of zebrafish melanocytes to understand cell biology and disease, the transcriptome-wide characterization of genes expressed in zebrafish pigment cells would be a significant resource.

In mammalian systems, melanocytes are the only neural crest-derived pigment cell type found in the dermis. In contrast, several neural crest-derived pigment cells are found in zebrafish and other poikilotherms, including reflective iridophores [5]. Several requirements for iridophore development from the neural crest are known [5], [26], [27]. However, it is unknown if markers of neural crest identity persist in iridophores following development, and whether these markers are shared by other neural crest-derived pigment cells, such as melanocytes. A further question in iridophore biology is how the guanine-based pigment is produced [28], [29]. Zebrafish bearing mutations in the *de novo* purine synthesis enzymes *gart* and *paics* have iridophore defects, indicating purine synthesis is important for iridophore pigmentation [30]. Identifying a possible mechanism by which iridophores produce an abundance of guanine for pigment formation while maintaining adequate supplies of purines for DNA and RNA production will be informative for cell biology.

Another pigment cell type shared by mammalian and poikilothermic vertebrates is the retinal pigmented epithelium (RPE). The RPE is a group of melanin-producing cells found in the vertebrate eye. The RPE develops from the eye primordium, and is continuous with the layer of cells that forms the iris [31]. The RPE is critical for eye development and retinal health. It provides trophic support and recycles wastes from the photoreceptors of the retina [32]. The RPE forms part of the blood-retina barrier, providing the eye with an immune-privileged status [33]. Defects in the RPE contribute to diseases such as macular degeneration and retinitis pigmentosa, which result in vision problems [34], [35]. Previous descriptions of gene expression in the RPE of zebrafish, chicken, and human have elucidated many of the genes playing roles in RPE biology [36]–[38]. Many of these genes are responsible for producing melanin, and defects in melanin production are often associated with reduced visual function [39]. However, it is unknown whether RPE and melanocytes use different pathways of melanin production, or if they are essentially identical. This information would be useful for understanding RPE biology, and would also inform future examinations of regulatory control for genes expressed in one or both cell types.

In order to facilitate understanding of these pigment cells, we developed a robust method to isolate these three pigment cell types from zebrafish embryos, followed by mRNA sequencing and transcriptome analysis. This purification procedure relies on the inherent densities of melanin and guanine-filled cells; hence it can be used without other complicated lineage markers. Here, we report the co-enrichment and cell-type specific gene expression profiles of melanocytes, iridophores, and RPE from embryonic zebrafish. While RPE and iridophores do not exhibit significant overlap of enriched gene expression, our analysis reveals considerable overlap among pairs of pigment cell types indicative of their common origin or function. Genes enriched in both the melanocyte and RPE lineages contain genes in the melanin-production pathway, and suggest a more complete picture of melanin-producing machinery is contained within this set. Similarly, expression co-enrichment in melanocytes and iridophores are reflective of their neural crest origin, and suggest genes in this set may be specific to neural crest identity. Furthermore, this is the first characterization of the iridophore transcriptome. We found that iridophores specifically upregulate the guanine portion of *de novo* purine synthesis, as well as specific enzymes from other metabolic pathways that aid in producing the iridophore guanine-based reflective pigment. In addition to the analysis presented here, this procedure and accompanying data provide a significant resource for further biological discovery in pigment cells.

## Materials and Methods

### Zebrafish Strain and Sample Collection Time Points

This study was carried out in accordance with the Washington University Animal Use Committee guidelines under approved protocol #20110236. Zebrafish were reared and bred according to standard protocols [40]. The fish used in this study were homozygous for a temperature sensitive allele of micropthalmia transcription factor (*mitfa^vc7^*) [41]. This mutant facilitated the collection of RPE, as *mitfa* is not required for RPE development in zebrafish [41]. Melanocytes, iridophores, and RPE develop normally at 25°C in *mitfa^vc7^.* When held at 32°C, the neural crest-derived melanocytes do not develop, but RPE and iridophores develop normally. All melanocyte and iridophore samples were incubated at 25°C prior to collection. We note the possibility that *mitfa* may be partially compromised by aberrant splicing products in this mutant at permissive temperatures, but following development at 25°C, melanocyte numbers, morphology, and pigmentation are indistinguishable from wild-type zebrafish [41]. Embryos used for RPE samples were incubated at either 25°C or 32°C until the equivalent of 3–5 days at 28.5°C, as indicated in Table S1 [42]. It should be noted that at stages later than 5dpf, choroidal melanocytes may be present adjacent to the RPE. Only one of the five RPE cDNA libraries was prepared later than 5dpf, as indicated in Table S1. We did not have separate markers to identify contamination from eye-associated neural crest-derived melanocytes, or choroidal melanocytes in our RPE preparations. If present, we expect the contribution to total gene expression in the RPE samples to exhibit high variance, and found to be insignificant by Student’s T-test upon comparison to the other cell types. However, we find the later stage RPE library to be highly correlated overall with the earlier stages, indicating that extensive contamination from choroidal melanocytes is unlikely (r = 0.92, Figure S1a). It was a further possibility that many large changes in gene expression would be present in RPE samples held at restrictive and permissive temperatures for *mitfa*. We found that samples held at the low and high temperatures exhibited a high degree of similarity (r = 0.89), indicating that most genes are not significantly different at the two temperatures (Figure S1b). We also expect some genes to change during development between 3 and 5 days post fertilization. Upon inspection of melanocyte samples prepared at several time points, we find correlations increase with increasing developmental age of the embryos (Figure S2). We do not have the statistical power to confidently track developmental changes in gene expression within specific cell types, but we make the data available for all individual libraries to facilitate further investigation (Table S2).

### Cell Dissociation and Pigment Cell Enrichment

**Figure 1:**
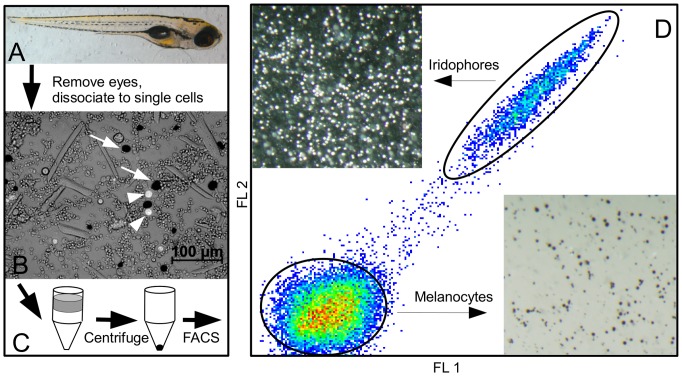
Purification procedure for melanocytes, iridophores, and retinal pigmented epithelium. Zebrafish are grown to the desired time point; shown in (A) is a six day old fish. (B) Fish are dissociated to a single cell suspension; black melanocytes (arrows) and reflective iridophores (arrowheads) are visible as a small percentage of all cells. (C) Cells are placed atop a Percoll density gradient and centrifuged. (D) The resulting cell pellet is resuspended and analyzed by FACS. Shown is a characteristic FACS plot demonstrating the relative positions of melanocyte and iridophore gates (ovals). Sorted iridophores are shown on the upper left of (D) under incident light. Sorted melanocytes are on the lower right using trans-illumination.

Figure 1 depicts the general procedure for pigment cell purification. Fish were anesthetized with Tricaine, rinsed with Ca-, Mg- DPBS (Sigma, D8537), and immersed in 100 mL TrypLE Express (Invitrogen, 12604039) per 1000 fish. Fish were incubated at 37°C and shaken at 100rpm for 15–20 minutes, followed by trituration with a Pasteur pipette to remove eyes from larva. After separation of eyes and larva, each group was placed in TrypLE Express and shaken at 100rpm at 37°C for 1–1.5 hr. Dissociated cells were filtered through a 120 uM screen into 50 mL tubes. Remaining intact tissue was triturated 10–20 times, and again filtered through a 120 uM screen into the dissociated cells. Dissociated cells were pelleted in a swinging bucket rotor (Eppendorf 5810 R) at 500 relative centrifugal force (rcf) for 5 minutes at 4°C, then resuspended in 1 mL cold isotonic Percoll (Sigma, P1644) by gentle pipetting. Isotonic Percoll was prepared by mixing 1 part 10X PBS with 9 parts Percoll. Resuspended cells were transferred to 1.6 mL Eppendorf tubes and spun at 2000rcf for 5 minutes at 4°C in a swinging bucket rotor for isopycnic separation. Pigment cells in the pellet were then resuspended in 400 µL of ice cold DPBS with 2% fetal calf serum (FCS), and placed onto preformed Percoll density gradients. Preformed gradients were prepared via centrifugation of 1 mL aliquots of isotonic Percoll in 1.6 mL tubes at 10,000rcf for 15 minutes at 4°C in a fixed angle micro-centrifuge (Eppendorf 5415 R). Tubes containing preformed Percoll gradients with overlying cell suspensions were centrifuged in a swinging bucket rotor at 2000rcf for 10 minutes at 4°C. Following centrifugation, overlying Percoll was aspirated, leaving the final 100 µL containing the pigment cell pellet. Cells were resuspended with 50 µL of cold DPBS with 2% FCS and transferred to a clean 1.6 mL tube containing 500 µL of cold DPBS with 2% FCS, and kept on ice until mRNA extraction or FACS.

### FACS

We used the inherent properties of the pigmented cells to perform Fluorescence-Activated Cell Sorting (FACS). Following resuspension in 500 µL cold DPBS with 2% FCS, the enriched cell populations were screened through a 30 uM cell filter (Partec, 04–0042–2316). Cells were analyzed and sorted with a Dako MoFlo cell sorter using a 120 uM nozzle at a drop drive (DD) frequency of 22390 Hz. Cells were illuminated using a 488 nm laser. Cells were gated on two attributes to separate cells from each other and from cellular debris. Cellular debris was detected using forward and side scatter, selecting against the smallest particles (∼1 µm or less). Cells were sorted based on detection using 510–530 nm and 575–595 nm filters, corresponding to FL1 and FL2 in Figure 1D, respectively. When excited by the 488 nm laser, the autofluorescence of iridophores is clearly detectable in these channels as a group of cells extending at a 45 degree line in the upper right quadrant. Melanocytes and RPE do not autofluoresce with this intensity when excited by the 488 nm laser, and cluster at the lower left of the FACS plot. Cells were collected into ice-cold DPBS with 2% FCS and kept on ice until mRNA extraction.

### mRNA Extraction, cDNA Synthesis, and Illumina Library Preparation

**Figure 2:**
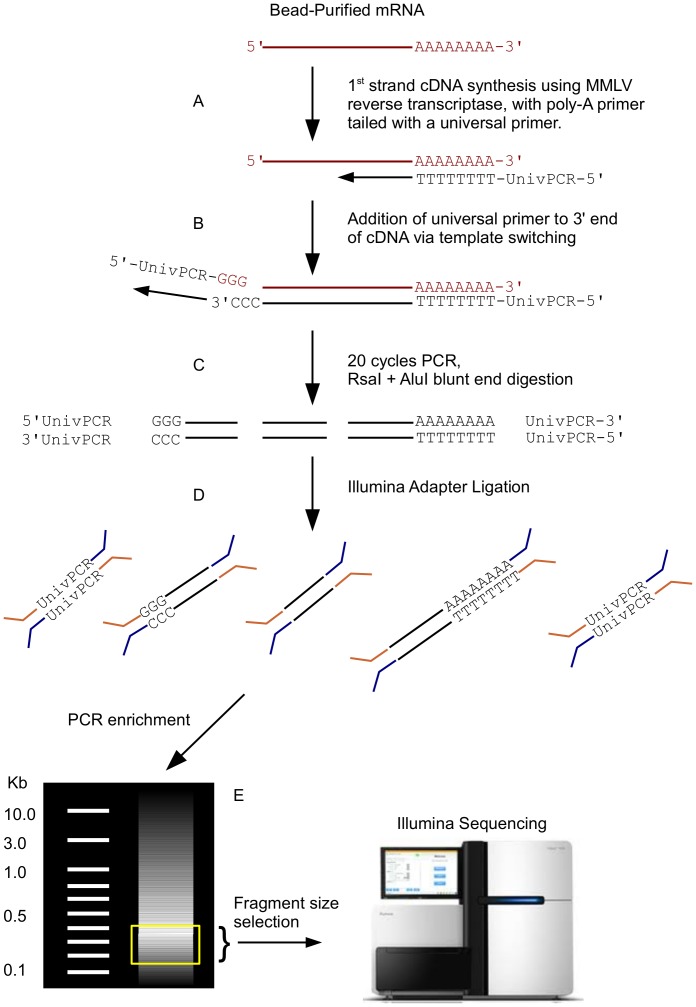
Schematic of cDNA library preparation. PolyA-selected mRNA (in red) is reverse transcribed using a polyT primer tailed with a universal primer (A). See Table S3 for primer sequences. MMLV reverse transcriptase adds cytosines to the 3′ end of the 1st strand cDNA (in black), allowing for template switching and addition of the 3′ universal primer (B). PCR amplification of the library is followed by RsaI and AluI enzymatic digestion of cDNAs (C), followed by the standard Illumina library preparation steps of end-repair, a single adenine addition, Y-adapter ligation (D), PCR enrichment, and size selection (mock gel shown in E with yellow box indicating area of gel removed for DNA extraction), prior to flowcell generation and sequencing.

Pigment cell cDNA library construction was as follows. For mRNA extraction the Dynabeads® mRNA DIRECT Kit (Invitrogen) was used per manufacturer’s instructions. Following mRNA elution from the Dynabeads, first strand cDNA synthesis was performed using MMLV reverse transcriptase (Clontech) using an anchored polyT primer tailed with a universal primer sequence (See Table S3 for primer sequences and Figure 2 for pigment cell cDNA library construction overview.) A universal primer sequence was also added to the 3′ end of the first strand by template switching, allowing for PCR-amplification of the resultant cDNA [43], [44]. Following PCR amplification using the high fidelity polymerase LA Taq (TaKaRa, PCR cycle: 95C for 1 minute, followed by 20 cycles of 98C for 25 seconds, 60C for 1 minute, 68C for 20 minutes), cDNA was digested with AluI and RsaI restriction enzymes (NEB). Blunt-end enzymatic fragmentation of cDNA was used instead of sonication and gel extraction to minimize loss of sample material and eliminate the end-repair step of Illumina library preparation. Since this reduced representation strategy might miss short cDNAs that lack both restriction sites, we sought to avoid this by including enzyme recognition sites within the cDNA amplification primers. This allows for the inclusion of short cDNAs in our libraries. Standard Illumina library preparations followed, performed by the Genome Technology Access Center (GTAC) at Washington University in St. Louis (http://gtac.wustl.edu). In brief, a single A was added to the 3′ end of each strand, Y-adapters ligated, and library enrichment PCR performed, followed by gel extraction size-selection for fragments ranging from 200–400 base pairs in length. Illumina library construction of pooled 3dpf embryos was performed by GTAC from total RNA extracted with Trizol reagent as previously described [45]. No PCR amplification of whole embryo cDNA was performed prior to Illumina adapter ligation and library enrichment. Sequencing was performed on the GAIIX and HiSeq 2000 Illumina platforms. Technical sequencing replicates of the same libraries on separate lanes were essentially identical (Figure S3).

### Sequence Analysis

We used Novoalign (www.novocraft.com), to assign resultant expression sequence tags to a customized non-redundant database of cDNA sequences consisting of 25,102 known and predicted genes (Table S4). Initial inspection of the NCBI zebrafish mRNA database of 28,286 zebrafish cDNAs (ftp://ftp.ncbi.nih.gov/refseq/D_rerio/mRNA_Prot/) via an all-by-all BLAST search revealed multiple nearly identical sequences for cDNAs that would confound the unambiguous assignment of sequence tags. In order to generate a non-redundant cDNA database we selected single representatives for each gene as follows. In instances where a cDNA in the database resulted in a BLAST hit of greater than 94% identity to more than 70% the length of another transcript, we excluded the smaller of the two cDNAs. The resultant non-redundant zebrafish cDNA database contained 25,102 unique gene records. We further analyzed our non-redundant database by mapping all cDNAs onto the UCSC zebrafish browser (ZV9). Manual examination of 6 Mbs arbitrarily chosen along chromosome 13 revealed 130 annotated genes (combined RefSeq and Ensembl gene tracks). Of these, our non-redundant database identified 126. The 4 genes not represented in our database included 3 5 S ribosomal RNA genes and ENSDARG00000086970, an annotated gene with a predicted ORF but no clear orthology to other species. Thus, this test shows that our method to generate a non-redundant cDNA library results in a database that identifies ∼99% of annotated genes (excluding ribosomal RNA genes). Furthermore, manual examination of our non-redundant library mapped onto this 6 Mb of chromosomal sequence revealed 10 sequences that were not annotated as RefSeq or Ensembl genes. Comparison to repeat tracks on the UCSC browser revealed that most (8/10) of these unannotated matches identified ORFs from repetitive, or retrotransposon DNA. It is not clear how these sequences were initially included in the NCBI cDNA database.

In summary, our efforts have generated a non-redundant zebrafish cDNA database that identifies most (∼99%) of annotated genes, and includes 7% (10/126) records of dubious utility, but would tend not to confound RNA-seq analysis such as reported here. Sequencing results for each cDNA library are summarized in Table S1. For each cDNA library, the number of tags aligned to each gene was normalized by the length of the gene and the total number of uniquely aligning reads for that library using custom Perl scripts (reads per kilobase of cDNA per million mapped reads - RPKM [45]. Statistical calculations were performed in R (www.r-project.org). All sequencing data used in this study are publicly available at NCBI’s GEO database under accession GSE46387 (http://www.ncbi.nlm.nih.gov/geo/query/acc.cgi?acc=GSE46387).

### Quantitative RT-PCR

Primers used for expression analysis are listed in Table S3, designed with NCBI’s Primer-BLAST to be separated by at least one intron (http://www.ncbi.nlm.nih.gov/tools/primer-blast/). Expression data was normalized relative to beta actin for each sample, using cDNA produced as described above, without PCR amplification. Q-PCR was performed in a Perkin-Elmer thermocycler with the following conditions: 95C 1 min, 98C 20 sec, 60C 1 min, repeated for 40 cycles.

## Results and Discussion

### Purification of Melanocytes, Iridophores, and RPE from Whole Embryos to Generate Cell-specific Gene Expression Data

At three days post fertilization (dpf), melanocytes, iridophores, and the RPE are readily visible in zebrafish. Melanocytes are extensively dendritic, and identifiable due to the presence of black melanin. Iridophores are round reflective cells, easily seen with a microscope using incident light. The RPE is present in the eyes as a hexagonally packed layer of melanized cells. However, the percentage of melanocytes, iridophores, and RPE compared to all other cells in the fish is less than 1%, making cell-specific gene expression analysis from the whole organism difficult. Aided by a previously reported method using density gradient centrifugation to isolate melanocytes from the caudal fins of Blackmoor goldfish [20], we developed a simple procedure to rapidly purify these pigmented cells from zebrafish embryos. This procedure relies on the inherent densities of the melanin-filled melanocytes and RPE, and the guanine-filled iridophores. In brief, cells are enzymatically dissociated from intact fish, enriched for pigment cells via density gradient centrifugation, and sorted by flow cytometry using the autoflourescent property of iridophores (Figure 1). The other pigment cells in zebrafish, the pteridine-containing xanthophores, do not pass through the density gradient and are not isolated by this method. Following cell purification, mRNA is isolated and Illumina libraries are constructed from cDNA (Figure 2). We purified melanocytes, iridophores, and RPE as described above and performed mRNA sequencing for 11, 5, and 5 independently isolated samples, respectively. In order to confirm the quality of our cell specific libraries, we assembled a list of genes known to be expressed in melanocytes, iridophores, and RPE. When we compared expression of these genes in our cDNA libraries to this control list, we observed expression corresponding to each cell type (Table 1). Furthermore, we also found good correlation for fold changes determined by qPCR of independently prepared biological samples and those determined by our RNA-seq data, indicating our RNA-seq fold change calculations between pigment cell types are realistic values (Figure S4). For instance, *ltk*, known to be a marker of iridophores, is highly enriched in iridophores compared to melanocytes (red square with x in Figure S4) [27]. Similarly, we find *dct*, a known requirement for melanogenesis, to be highly enriched in melanocytes and RPE relative to iridophores. Interestingly, we also find *rpe65a*, well-known to be expressed in RPE, to also be expressed by melanocytes. This is not entirely surprising, as RPE65 is known to be present in keratinocytes, melanocytes, and melanoma, in addition to the RPE [46], [47]. Thus, using this method, cDNA from melanocytes, iridophores, and RPE can be generated concomitantly from thousands of whole zebrafish embryos in a single day. The development of a fast and robust method for purifying these pigment cells greatly simplifies the production of multiple biological replicates needed for informative analysis of high-throughput expression data.

**Table 1:** Candidate control genes are differentially expressed.

	RPKM Counts			Student’s T-test p-Values		
	Melanocyte	RPE	Iridophore	Melanocyte vs. RPE	Melanocyte vs. Iridophore	RPE vs. Iridophore
**Melanocyte Genes**						
*gch2*	54.25	9.81	1.58	0.007	0.002	0.113
*mlphb*	187.84	7.96	1.39	0.004	0.003	0.251
*kita*	3.78	0.39	0.02	0.019	0.011	0.076
**Melanin Synthesis**						
*pmela*	15177.25	4244.20	148.90	0.051	0.011	0.031
*dct*	14134.12	6406.98	402.47	0.025	0.000	0.028
*tyrp1b*	12354.09	3769.95	200.99	0.026	0.004	0.009
**Iridophore Genes**						
*atic*	7.60	9.63	467.16	0.664	0.010	0.010
*ednrb1*	4.44	1.72	28.63	0.110	0.018	0.013
*ltk*	0.02	0.20	4.02	0.138	0.002	0.002
**Neural Crest**						
*sox10*	7.57	1.76	13.00	0.010	0.078	0.005
*foxd3*	3.03	0.35	6.41	0.012	0.237	0.063
*snai2*	4.26	0.79	4.08	0.016	0.904	0.020
**RPE Genes**						
*pax6a*	10.04	47.48	0.73	0.098	0.004	0.055
*nr2e1*	0.75	2.41	0.05	0.189	0.041	0.086
*myo7ab*	1.89	3.32	0.15	0.050	0.001	0.003

Selected genes indicative of pigment cell identity or shared functions are shown.

### Analysis of Gene Expression in Melanocytes, Iridophores, and Retinal Pigmented Epithelium

We first aimed to use our RNA-seq data to identify the total number of genes expressed in each of the pigment cell types. To eliminate low-level background expression, we applied a baseline expression threshold of 1 read per kilobase of transcript per million reads (1 RPKM). We chose this threshold based on the ability to detect expression of known pigmentation and neural crest genes. For example, we found *mc1r*, *kita*, and *foxd3* to have RPKM values in melanocytes of 2.2 and 3.8, and 3.0, respectively (Table 1). Furthermore, we detected more than 95% of the genes expressed at 1 RPKM with a sequencing depth of approximately one million reads per library, which established our sequencing depth threshold (Figure S5). Using this baseline threshold of 1 RPKM, we found 8,472 genes are expressed by melanocytes, 8,096 by iridophores, and 9,053 by the RPE (See Table S5 for the averaged RPKMs and T-test values). To put this into perspective, we also aligned over 700 million cDNA tags generated by the Stemple laboratory [48] from a variety of embryonic stages and adult tissues. We found this mixed data set revealed expression of at least 1 RPKM for 20,548 gene entries from our database of 25,102 unique coding sequences (Not shown). These results indicate our non-redundant database is a reasonable approximation of the protein-coding transcriptome in zebrafish, and conclude that 30–40% of all genes are appreciably expressed (>1 RPKM) by these specific cell types.

### Melanocyte, Iridophore, and RPE Gene Expression is Correlated Compared to Whole Embryos

Having obtained gene expression data for these pigment cells, we set out to identify signatures of pigment cell functions from the data sets. We reasoned that genes co-enriched among pigment cell types would indicate shared pigment cell functions. As an initial assessment of the similarity of gene expression between melanocytes, RPE, and iridophores, we applied Pearson’s Product-Moment Correlation test to the datasets. Because correlation values can be artificially skewed by outlying data points [49], we calculated the average correlation from 24,102 overlapping windows of 1000 genes, sorted by increasing whole embryo expression (Figure S6). Using this metric, we found the melanin-producing melanocytes and RPE were the most highly correlated (r = 0.90). The neural crest-derived melanocytes and iridophores were modestly correlated (r = 0.52). RPE and iridophores were also modestly correlated (r = 0.49). We expected the correlation to be dramatically lower when comparing a single purified cell type to whole embryos than when comparing two purified pigment cell types to each other. We thus determined the correlations of each pigment cell type to mRNA-seq data from whole 3dpf zebrafish. By this general assessment, melanocytes, RPE, and iridophores are distinct from whole embryos, with average correlation values less than 0.02. Presumably, this low value (0.02) reflects the baseline correlation component from expression of housekeeping genes shared by all cells, and greater values (0.4–0.9) reflect shared specific gene expression among the pigment cell types.

### Gene Expression Indicative of a Pigment Cell Identity

These correlations described above suggest there are genes enriched in all three pigment cell types that are generally indicative of pigment cell identity. It is not clear *a priori* which genes should be shared by different pigment cell types. In order to construct an informative set of genes for this purpose, we searched for those that are expressed within a 2-fold change of each other at a minimum of 4 RPKM, and at least 100-fold greater than whole 3dpf embryos. We found 28 genes fulfilled these criteria (Table 2). Remarkably, 4 of the top 5 highest expressed genes on this list are ribosomal proteins. Pigment cell enrichment of ribosomal components is not surprising, considering several mouse coat color mutants are ribosomal proteins, although they are not the ribosomal components enriched here [50]–[53]. We also find the zebrafish *albino* gene (*slc45a2*) to be among the highest expressed genes in each of these pigment cell types [54], [55]. The expression of *slc45a2* in iridophores is interesting, considering *albino* fish are not reported to have an iridophore defect. One possibility is that SLC45A2 performs a common role for organelle pH homeostasis in pigment cells. However, *in situ* analysis reveals no enriched expression in xanthophores, suggesting that *slc45a2* expression is not shared by all pigment cells [55]. This list of co-expressed genes also contains several other unexpected members, including the Jak-STAT cytokine receptor *crfb5*, and the BAX-inhibitor protein *ghitm*. It is not clear what roles these genes play in pigment cells, but this list provides a starting point for understanding their shared functions in pigment cell biology.

**Table 2:** Shared gene expression among melanocyte, RPE, and iridophore.

Gene	Melanocyte	RPE	Iridophore	Embryo	Notes
*rpl26*	22354.26	19692.74	23528.06	87.23	Ribosomal protein
*rps17*	12677.82	10420.73	16940.17	59.88	Diamond-Blackfan Anemia [90]
*rps2*	12516.82	9306.57	12379.31	89.39	Ribosomal protein
*rpl27a*	6506.97	5852.65	8537.27	48.50	Ribosomal protein
*slc45a2*	4415.88	2376.16	2801.48	0.20	*albino* locus [54], [55]
*rps26l*	2338.29	2362.00	2596.78	23.08	Ribosomal protein
*ppp1r21*	263.06	227.24	202.38	1.77	Protein phosphatase
*crfb5*	237.93	329.44	301.82	1.19	Jak-STAT cytokine receptor
*LOC100535047*	217.00	212.13	135.05	0.40	Uncharacterized
*dhdh*	201.46	185.72	161.64	1.52	dihydrodiol dehydrogenase
*cyhr1*	150.55	157.36	138.95	0.87	cysteine/histidine-rich 1
*igf2bp2b*	133.81	97.93	128.56	0.75	mRNA-binding protein
*ghitm*	133.71	119.78	162.37	0.99	BAX inhibitor protein family
*her9*	126.24	85.17	120.90	0.71	NOTCH pathway
*fam168a*	89.26	78.21	73.66	0.44	Chemoresistance [91]
*comtb*	81.11	55.05	70.61	0.32	Dopamine degradation
*fkbp3*	75.26	59.48	79.66	0.01	Rapamycin binding protein
*mtbl*	65.94	36.58	48.31	0.32	Heavy metal resistance [92]
*pard3b*	53.47	62.57	46.53	0.38	Cell-cycle
*hbp1*	41.93	50.61	52.08	0.12	SOX-TCF-HMG family transcription factor
*zgc:158345*	31.67	43.92	59.38	0.31	Tyrosine phosphatase, PTEN C2 domain
*ccdc85al*	31.26	34.99	40.96	0.13	Uncharacterized, coiled-coil protein
*grma*	17.17	18.26	28.83	0.10	Glutamate receptor, GPCR
*mbd2*	16.57	16.03	16.31	0.13	Binds methylated DNA
*triobpl*	14.28	16.86	12.85	0.01	TRIOBP-like, actin organization [93]
*rnd2*	14.23	10.66	12.26	0.00	Rho GTPase, neurite branching [94]
*LOC100334991*	10.17	7.87	10.08	0.03	Uncharacterized
*zgc:136564*	5.94	9.00	5.30	0.04	C9orf64 homologue, unknown function

Shown are RPKM values for genes co-enriched among the three pigment cell types at a level 100-fold greater than whole embryos, within a 2-fold change of each other, with a minimum RPKM of 4.

### Gene Expression Indicative of Cellular Function or Developmental Origin

We were also interested to identify genes that were enriched in only two pigment cell types that would reveal shared function or developmental origin. For this analysis, we filtered for shared expression of genes in two cell types at least 2-fold over the third cell type, where differences exceeded a significance of p<0.05. Upon validation of expression differences via qPCR between pigment cell genes and whole embryos, we found a systematic bias of overcalling the fold changes between pigment cell values and whole embryos (Figure S7). Based on this result, we also required an 8-fold greater than embryo expression threshold for co-enrichment and cell-specific genes, as discussed below. Using these criteria, we found 214 genes were enriched in both melanocytes and RPE but not iridophores (Table S6), and 62 genes enriched in melanocytes and iridophores but not RPE (Table S7). Given that the RPE and iridophores do not share a pigment-type production or developmental origin, we expected fewer genes to be co-enriched in these cells, but not expressed by neural-crest melanocytes. Only one gene, *alcama* (also *dm-grasp* or *neurolin-a*), the target of the zn-5/8 monoclonal antibody [56], [57] was enriched in both RPE and iridophores when compared to melanocytes and whole embryos (Table S8). This is consistent with the notion there are few specific functions shared only between iridophores and RPE. It is intriguing that *alcama* has been found to mediate endothelin 1 signaling in cartilage development [58]. Relatedly, endothelin receptor (*ednrb1*) signaling is required for iridophore development in zebrafish [5]. It will be interesting to know whether *alcama* mutations have functional consequences in the iridophore or the RPE. Therefore, we suggest these lists of co-expressed genes are likely enriched for common metabolic functions, in the case of melanocytes and RPE, or developmental origins, in the case of melanocytes and iridophores.

### Melanocyte and RPE Co-Expression

Due to their shared function of producing melanin and melanosomes, we expected many genes to be shared between melanocytes and the RPE when compared to iridophores. We found 214 genes that fit our parameters for shared enrichment (Table S6). As expected, there are many genes present involved in melanin synthesis and melanosome biogenesis, including *pmela*, *dct*, *tyrp1b*, *tyrp1*, *pah*, and *slc24a5*. There are several transcription factors in this enrichment group, including three forkhead box (*foxo1b*, *foxp4*, and *foxg1b*) and five homeobox-containing transcription factors (*hmx1*, *hmx4*, *otx1a*, *otx2*, and *otx5*). Although not required for RPE development in zebrafish, *mitfa* is expressed in the RPE at a relatively high level, consistent with the reported ability of *mitfa* to promote pigmented fate in zebrafish retinas [59]. However, as we find most of the other transcription factors co-enriched in melanocytes and RPE are expressed at lower levels, it is interesting to speculate that one or more of these factors are able to compensate for *mitfa* specifically in the RPE. Thus, this co-enrichment set may identify a more comprehensive list of genes for melanocyte and RPE identity and melanosome biogenesis.

An additional finding apparent from the 38 most highly expressed genes co-enriched in melanocytes and RPE is that iridophores also express these genes, albeit at a much lower level (Table 3). The close developmental lineage relationship between melanocytes and iridophores suggests a possible “leakiness” of specificity that may result in weak expression of melanocyte genes in iridophores. This possibility is supported by the observation that the *parade* zebrafish mutant contains pigment cells with both melanocyte and iridophore characteristics [22].

**Table 3:** Shared gene expression among melanocyte and RPE.

Gene	Melanocyte	RPE	Iridophore	Embryo	Notes
*pmela*	15177.25	4244.20	148.90	0.57	*Silver* mouse, *fading vision* zebrafish [95]
*dct*	14134.12	6406.98	402.47	0.65	Melanin synthesis
*tyrp1b*	12354.09	3769.95	200.99	1.13	Melanin synthesis
*tyrp1*	2546.87	1474.07	71.46	0.03	Melanin synthesis
*rlbp1b*	1181.62	1212.86	48.19	0.26	Retinaldehyde binding protein
*mitfa*	1122.44	448.53	16.22	0.00	Melanocyte master regulator
*pah*	1081.84	485.25	35.79	23.60	Melanin synthesis
*stra6*	703.09	539.15	32.81	3.00	Retinol metabolism
*rgra*	300.56	504.34	27.16	0.21	GPCR
*msnb*	254.08	217.35	11.10	0.45	FERM family, RDX homologue
*fam213ab*	217.86	160.16	12.32	0.28	Antioxidant enzyme
*rbp1a*	148.55	137.02	8.74	1.79	Retinol binding protein
*slc24a4a*	141.08	89.64	7.63	1.26	Neural crest and RPE expression [96]
*zgc:114181*	122.41	128.26	6.71	0.58	Putative CNDP1 homologue
*mab21l2*	120.20	176.48	2.98	0.82	Eye development [97]
*LOC100004225*	105.78	52.37	3.46	1.32	Uncharacterized
*lratl*	105.09	96.40	3.52	0.39	Lecithin retinol acyltransferase
*oca2*	102.56	56.96	1.63	0.07	*Pinkeyed-dilution* mouse [98]
*kif21al*	96.64	104.62	4.16	0.82	Kinesin family member, *LOC100537698*
*dhrs11al*	95.71	130.88	6.86	8.45	Dehydrogenase/reductase SDR family
*cadm3*	87.46	116.23	4.88	0.12	Nectin family cell adhesion protein
*cdh2*	86.69	99.72	6.17	1.10	Cell adhesion
*slc24a5*	83.81	34.68	1.56	0.00	*golden* locus [99]
*s1pr1*	83.14	74.37	4.78	1.20	Rhodopsin family GPCR
*ctgf*	62.15	62.19	4.20	0.32	Secreted mitogen
*fads6*	49.52	84.55	2.76	0.08	Fatty acid biosynthesis
*foxp4*	39.96	84.03	1.78	0.25	Neuronal arborization maintenance [100]
*foxg1b*	39.71	64.02	2.38	0.10	Forkhead box transcription factor
*kif21al*	37.47	58.20	3.21	0.37	Kinesin family member
*abcg2d*	34.72	48.81	1.58	0.00	*white* family member [101]
*LOC100149324*	33.08	31.15	1.55	0.14	Uncharacterized phospholipase
*efcab4b*	30.89	38.65	1.71	0.00	Calcium sensing GTPase
*col11a1a*	27.39	27.11	1.22	0.75	Collagen alpha chain precursor
*rdh13*	24.30	31.04	0.81	0.00	Retinol dehydrogenase
*cam4l*	23.38	37.54	1.34	0.29	Cell adhesion molecule 4-like
*srcrb4l*	21.14	20.88	1.18	0.80	Scavenger receptor Cys-rich group B-like
*col4a5*	20.86	29.39	1.69	0.49	Type IV collagen
*dao.2*	20.27	22.39	1.23	1.39	D-amino-acid-oxidase

RPKM values for genes co-expressed in melanocyte and RPE at least 10-fold greater than iridophores and whole embryos, with a minimum of 10 RPKM.

### Melanocyte and Iridophore Co-Expression

Because of the common developmental origin from the neural crest for melanocytes and iridophores, we speculated that neural crest-specific gene expression would be readily identifiable. We found 62 genes that were significantly upregulated in melanocytes and iridophores over RPE (Table S7). A list of the 15 genes expressed at least 5-fold greater than RPE and 10-fold greater than whole embryos is shown in Table 4. Included in this group are several well-known regulators and markers of neural crest and pigment cell development, including the transcription factors *sox10* and the expressed repetitive element *crestin* [60]. The well-known pigmentation gene *mc1r* is also expressed in iridophores and melanocytes. Also included is the cell adhesion molecule *pcdh10a*, which is expressed by migrating zebrafish neural crest cells, and acts as a tumor suppressor in several cancers [61]-[63]. We also find a retinoic acid nuclear receptor subfamily member (*rxrga*) previously reported to be expressed in neural crest tissues [64]–[67]. Because we find this known set of neural crest genes in this co-enrichment list, other unknown markers of neural crest identity are likely to be present. For instance, several transcription factors are in this enrichment set not previously reported in neural crest, including the forkhead box transcription factor *foxo1a*, as well as the cell cycle regulator *cdk15*. Thus, this list of genes co-enriched in melanocytes and iridophores may more broadly identify markers of neural crest origin.

**Table 4:** Shared gene expression among melanocytes and iridophores.

Gene	Melanocyte	Iridophore	RPE	Embryo	Notes
*syngr2l*	86.584	50.745	5.728	1.471	Unclear function, transmembrane protein
*tuba8l3*	78.779	132.465	11.725	0.397	*puma* locus [102]
*pcdh10a*	59.639	54.800	5.143	0.423	Neural crest expression [64]
crestin	16.922	25.646	2.214	0.145	Expressed repetitive element [60]
*LOC559216*	14.652	27.212	1.350	0.689	Uncharacterized RhoGEF
*si:dkey-72l14.7*	11.657	40.247	1.004	0.000	Rhodopsin family GPCR
*cdk15*	8.492	6.475	0.291	0.112	Cyclin dependent kinase
*emp3l*	6.859	17.644	0.293	0.046	Tumor suppressor [103]
*lamb1b*	5.486	6.138	0.630	0.427	Laminin-type EGF-like domains
*zgc:158328*	4.355	8.553	0.776	0.004	Uncharacterized, EMI and vWBF domains
*opn5*	3.824	1.796	0.193	0.000	Rhodopsin family GPCR
*ppfia2*	3.521	3.859	0.443	0.037	Axon guidance [104]
*rab27bl*	3.263	13.331	0.278	0.118	Melanosome transport [105]
*mc1r*	2.182	3.958	0.076	0.000	GPCR for melanocyte stimulating hormone
*birc7*	1.818	2.960	0.233	0.000	Inhibitor of apoptosis protein [106]

RPKM values for genes expressed in melanocytes and iridophores at least 5-fold greater than RPE and whole embryos.

### Cell-Type Specific Gene Expression

We were also interested in determining gene expression specific to each cell type. Because melanocyte and RPE gene expression have been previously characterized, we do not discuss them in detail here, but present the data as the supplemental tables listed below [36]–[38], [68], [69]. For this analysis we required an expression level at least 2-fold over the other two cell types (p<0.05), as well as 8-fold greater expression than whole embryos. This filtering strategy resulted in a list of 108 genes specifically enriched in melanocytes (Table S9) and 24 in the RPE (Table S11). To independently validate these enrichment sets, we generated separate biological samples for each cell type and compared expression of selected genes from the enrichment sets via qRT-PCR. We observed good correlation of RNA-seq and qRT-PCR relative expression levels, indicating our RNA-seq data is a reliable indicator of the gene expression in these pigment cells (r^2^ = 0.75, Figure S4). These data will be useful for further studies of melanocyte and RPE biology.

### Iridophore Gene Enrichment

In contrast to melanocytes and RPE, the iridophore transcriptome has not previously been explored. Since iridophore produce a guanine-based pigment, rather than the melanin characteristic of melanocytes and RPE, we expected to find many genes to be specifically enriched in this cell type. This indeed turned out to be the case, with 346 genes passing our baseline threshold for enrichment (Table S10). Included in this enrichment list are several factors known to be important for iridophore development, including *ltk*, *ednrb1*, and *pnp4a* [5], [26], [27]. Also, in order to identify previously unreported genes that may play interesting roles in iridophores, we filtered our list to include genes expressed at least 30-fold greater than melanocytes and RPE, and 100-fold greater than whole embryos. Thirty genes met these criteria (Table 5). The third highest expressed gene on this list, *slc23l*, may act as a guanine transporter. In mammals, the SLC23 gene family has roles in transporting nucleobases, such as guanine, as well as vitamin C. It is not clear how this highly expressed iridophore gene identifies a unique role for vitamin C in the iridophore, but it is tempting to speculate a role in guanine transport, either to transport guanine into the cell, or perhaps to transport newly synthesized guanine into the reflecting platelet organelles. One surprise is the finding that *gpnmb* is highly enriched in the iridophore. Roles for GPNMB have been described for melanocytes, melanoma, and the pigmented iris in mammals, and it has been suggested to act both as a plasma membrane protein and a component of the melanosome [70]. Our finding that *gpnmb* is more highly expressed in the iridophore than the melanocyte raises the possibility of a similar function in iridophore reflecting platelet organelle biogenesis. Also in this iridophore-specific enrichment list are six uncharacterized genes. Their enrichment in iridophores may aid in identifying functions for these proteins. We speculate that many iridophore-enriched genes will be indicative of novel cell-specific biological functions.

**Table 5:** Iridophore enriched genes.

Gene	Iridophore	Melanocyte	RPE	Embryo	Notes
*ifi30l*	2138.17	6.55	27.16	1.23	Melanoma antigen processing [107]
*fhl3*	1003.29	4.64	20.97	1.12	Actin organization [108]
*slc23l*	592.05	10.64	9.70	1.19	Nucleobase transport [109]
*gpnmb*	423.58	1.02	5.28	1.70	Glycoprotein [70]
*LOC100538040*	396.38	1.52	9.30	1.85	Uncharacterized, likely GPI-linked glycoprotein
*LOC100334697*	330.19	1.06	4.78	0.09	Crystal protein-like
*tpd52l1*	252.61	5.69	7.75	1.02	Membrane trafficking [110]
*LOC100535932*	221.28	0.98	4.64	0.18	Uncharacterized
*pltp*	145.30	1.07	2.02	0.02	Lipoprotein metabolism [111]
*LOC795494*	127.94	0.43	2.17	0.11	Nucleotide metabolism domain
*LOC100330987*	99.01	0.56	1.80	0.01	Uncharacterized
*zgc:77375*	94.86	0.44	2.24	0.01	Haloacid dehalogenase
*slc25a38a*	94.77	1.10	1.63	0.46	Mitochondrial carrier protein
*LOC100534970*	81.87	0.65	2.48	0.18	Uncharacterized
*tmem179bl*	79.07	0.32	0.66	0.14	Transmembrane protein
*fkbp15*	32.47	0.55	0.51	0.31	Uncharacterized
*pcolcel*	30.46	0.31	0.22	0.00	Extracellular matrix protein
*si:ch211-38m6.6*	28.42	0.12	0.23	0.00	Major Facilitator Superfamily
*tagln3b*	24.88	0.13	0.74	0.06	Cytoskeleton-associated [112]
*alx4b*	24.81	0.10	0.24	0.08	Skull ossification [89]
*hsf5*	21.07	0.05	0.20	0.00	Transcription factor
*osbpl10*	18.67	0.26	0.55	0.08	Intracellular lipid receptor
*si:dkey-225f23.4*	14.27	0.14	0.13	0.00	Uncharacterized
*zgc:112054*	12.99	0.34	0.40	0.00	bZIP transcription factor
*slc52a3*	12.73	0.03	0.12	0.12	Riboflavin transporter
*cart1*	9.87	0.19	0.28	0.00	*ALX1* homolog
*myadm*	7.81	0.14	0.23	0.00	Myeloid associated differentiation
*znf831*	7.04	0.09	0.10	0.06	Zinc-finger double domain
*si:ch211-14k19.8*	5.80	0.06	0.09	0.00	Uncharacterized
*nfascl*	5.27	0.07	0.11	0.00	Fibronectin domain

RPKM values for genes expressed in iridophores at least 30-fold greater than melanocytes and RPE, and 100-fold greater than embryos.

**Figure 3:**
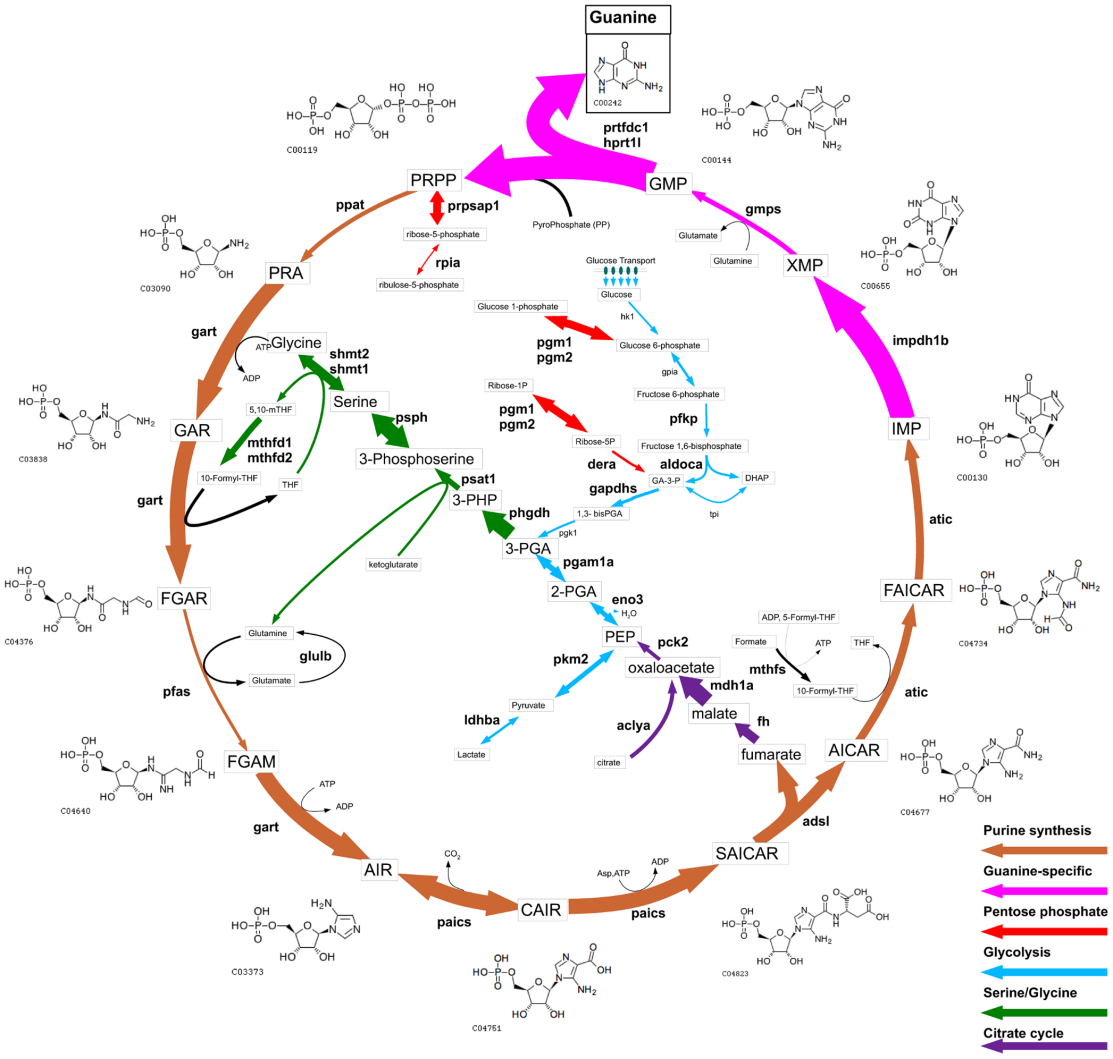
The guanine synthesis cycle is highly enriched in iridophores. Shown is a model for guanine production based on transcriptome data as given in Table 6. Genes that are statistically enriched compared to melanocytes are shown in bold, those not statistically different are in normal font. The arrow thicknesses correspond to the fold changes in iridophores relative to whole embryos. Chemical structures are from the KEGG Compound database.

Many of the genes previously known to be expressed by iridophores are components of the purine synthesis pathway, as iridophore pigment largely consists of stacks of guanine plates [30], [71]. Accordingly, we found a dramatic enrichment of enzymes comprising the pathway of guanine metabolism, from extracellular glucose import and glycolysis, through *de novo* synthesis and purine salvage (Table 6). When comparing iridophores to melanocytes and RPE, we find 5 facilitated glucose transporters, 7/11 steps of glycolysis, and 9/9 enzymes for *de novo* purine synthesis to be enriched. Given that iridophore pigment consists largely of guanine, one might expect the guanine pathway to be specifically upregulated. Consistent with this model, we found the split in the purine synthesis pathway at IMP to favor guanine production rather than adenine. The first guanine-specific enzyme, *impdh1b*, is expressed at a level 71-fold greater than melanocytes. In contrast, the first adenine-specific enzyme, *adssl*, is not significantly different from melanocytes or RPE, at 0.7-fold the level of melanocytes. Upon inspection of the known pathway of guanine production, we observed synthesis of guanine from GMP likely results in the recycling of 5-Phosphoribosyl 1-Pyrophosphate (PRPP), the rate-limiting substrate in purine synthesis (KEGG Pathway: dre00230). The enzyme responsible for this final step of guanine synthesis, *prtfdc1*, is enriched 198-fold over melanocytes (p<0.01). From our expression data, we suggest a model of guanine pigment production in iridophores that illustrates a cycle of guanine synthesis utilizing PRPP as a recycled carrier molecule (Figure 3). In this cycle, specific enzymes from glycolysis, the pentose phosphate pathway, serine/glycine metabolism, and the citrate cycle, are upregulated to coordinate the extensive guanine synthesis required for the reflective iridophore pigment.

**Table 6:** Guanine synthesis-related gene expression enrichment in iridophores.

	RPKM				P-Value	
Gene	Iridophore	Melanocyte	RPE	Embryo	Irid vs. Mel	Irid vs. RPE
*slc2a15a (GLUT5)*	48.55	0.16	0.73	0.28	7.66E-03	7.89E-03
*pgm2*	34.00	0.54	0.73	1.24	4.64E-03	4.68E-03
*dera*	3.67	0.98	0.39	2.76	3.40E-04	1.55E-04
*pfkp*	12.25	0.63	1.28	2.42	1.31E-02	1.53E-02
*aldoca*	6.22	0.04	0.00	0.83	2.50E-03	2.45E-03
*gapdhs*	1605.92	191.73	163.31	9.03	1.92E-02	1.79E-02
*pgk1*	281.34	146.41	125.46	38.36	9.05E-03	7.22E-03
*pgam1a*	587.26	216.58	158.84	32.46	1.13E-03	4.02E-04
*eno3*	1503.93	215.46	307.89	100.27	4.30E-08	4.09E-05
*pkm2a*	371.83	115.96	97.94	24.94	4.52E-04	2.83E-04
*ldhba*	440.62	74.53	44.46	72.72	2.70E-04	2.85E-04
phgdh*	1538.41	371.96	175.60	8.25	4.21E-03	2.22E-03
*psat1*	160.85	7.03	11.92	7.62	4.68E-03	3.85E-03
*psph*	2602.05	146.30	102.78	1.36	1.53E-03	1.38E-03
*shmt2*	86.02	5.37	5.92	2.83	9.83E-03	9.50E-03
*mthfd1*	177.66	7.98	8.41	13.99	2.71E-02	2.72E-02
*fh*	562.28	150.18	109.27	8.65	2.53E-02	1.81E-02
mdh1a*	3948.05	388.29	432.95	27.33	2.55E-05	1.48E-05
*pck2*	1.44	0.10	0.01	6.09	1.05E-02	8.50E-03
*aclya*	106.57	42.29	39.30	8.19	2.49E-03	1.72E-03
*rpia*	11.66	2.49	2.24	6.52	4.70E-03	3.89E-03
prpsap1*	95.27	35.48	24.12	3.13	3.13E-03	1.88E-03
*ppat*	49.23	2.49	3.72	7.89	4.22E-03	4.26E-03
*gart*	167.66	6.41	4.29	3.46	6.01E-03	5.72E-03
*pfas*	34.59	0.53	0.56	4.53	1.52E-03	1.51E-03
*paics*	1465.37	57.12	55.35	11.68	3.63E-03	3.50E-03
*adsl*	805.77	46.06	36.43	12.45	2.69E-03	2.38E-03
*atic*	467.16	7.60	9.63	18.34	1.01E-02	1.02E-02
*impdh1b*	816.19	11.45	21.98	1.06	1.30E-03	1.21E-03
*gmps*	102.41	3.86	5.14	6.35	1.15E-03	1.02E-03
prtfdc1*	309.88	1.56	3.10	0.00	1.39E-03	1.39E-03
*hprt1l*	21.31	0.25	1.13	15.99	7.28E-04	6.03E-04
*adssl*	0.73	1.04	1.75	6.82	2.61E-01	1.08E-01
*ak1*	87.12	228.27	149.74	40.66	4.24E-03	1.75E-01

RPKM values for enzymes related to guanine synthesis in iridophores. Enzymes are grouped with their commonly associated pathways. Rate-limiting enzymes from each of the specific pathways are indicated with an asterisk. Notably, *adssl* is the first adenine-specific gene in purine synthesis, and is not upregulated in iridophores.

Another question in iridophore biology is how the membranous platelets containing the reflective guanine crystals are formed. Our data suggests a likely contribution from ADP Ribosylation Factors (ARFs) and Rab GTPases. ARFs are a large family of ras-related GTPases that regulate membrane trafficking and organelle structure (For review see [72]). We find two ARF-related genes to be significantly enriched in iridophores compared to melanocytes and RPE, *arf6* and *arfip1*. Interestingly, we also found two members of the ras-related oncogene family to be enriched in iridophores; *rab27b* and *rab38*. Rab GTPases regulate many aspects of membranous vesicle formation and traffic [73]. In humans, Rab27 mutations cause hypopigmentation associated with the immunodeficiency disorder Griscelli syndrome type II [74]. In addition, the formation of COPI transport vesicles is known to be mediated by the interaction of GAPDH with RAB2 [75]. Phosphorylation of GAPDH occurs through the Src/PI3K/AKT pathway, often downstream of receptor tyrosine kinase activation [76]. We found a single receptor tyrosine kinase enriched in iridophores, *ltk*, which we have previously shown to be required for iridophore development [27]. It is thus interesting to speculate that combined roles exist for GAPDH in both producing reactive carbonyl species during glycolysis for guanine production, as well as in forming the organelles within which iridophore pigment is contained.

Iridophores are a relatively less-studied pigment cell than melanocytes and RPE, and aside from the requirements for *foxd3*, *ednrb1*, and *ltk*, not much is known regarding the transcriptional regulation of iridophore identity. One might expect a single master regulator of iridophore identity, analogous to *mitfa* in melanocytes, which is highly expressed, to be readily identifiable in our data. However, we did not identify a highly expressed single candidate for a master regulator of iridophore identity. Instead, we found many moderately expressed transcription factors with known mouse or human orthologues to be significantly enriched in iridophores. Of these, there are several candidates that stand out as possible regulators of iridophore identity. One member of the basic helix-loop-helix (bHLH) family of transcription factors is specifically enriched in iridophores, *tfec*. Known to be expressed in iridophores, TFEC forms hetero and homodimers with other bHLH members and can function as a transcriptional activator or repressor [77]–[79]. Interestingly, there is one other bHLH in enriched in iridophores when compared to melanocytes and RPE, but it did not meet the 8-fold requirement over whole embryos. This gene, *mycl1a*, is a paralog of the classic oncogenic protein MYC. A key component of iridophore identity is the upregulation of glycolysis and guanine synthesis enzymes. It is established that MYC upregulates glycolysis, DNA-synthesis, and nucleotide metabolism [80]–[82]. Another oncogene shown to regulate glycolysis and associated feeder pathways is Ets-1 [83]. The v-ets-erythroblastosis virus E26 oncogene homolog 1a (*ets1a*) is also highly enriched in iridophores. In mouse, *tfec* transcription is activated through multiple ets-binding domains in its promoter region, suggesting a conserved regulatory mechanism for ETS1A in *tfec* transcriptional regulation as well [84]. Further work will be necessary to determine whether TFEC and MYCl1A coordinate the upregulation of guanine synthesis enzymes in iridophores.

We also find five homeobox-containing transcription factors are enriched in iridophores: *gbx2*, *cart1*, *alx3 alx4a*, and *alx4b*. Known to have several roles in the embryo and an early specifier of posterior neural crest in *Xenopus*, gastrulation brain homeobox 2 (*gbx2*), is highly enriched in iridophores [85]. Remarkably, the other four homeobox transcription factors we identified are all aristaless-related; *cart1*, *alx3, alx4a*, and *alx4b*. Members of the Cart1/Alx3/Alx4 family of homeodomain proteins are known to regulate formation of skeletal elements in organisms ranging from sea urchin to mammals [86], [87]. In humans, mutation and haploinsufficiency of ALX4 are associated with skull ossification defects [88], [89]. It is not clear how these aristaless-related transcription factors might regulate iridophore identity, but recognizing their expression here will aid in understanding their function. Together, these iridophore-enriched transcription factors likely play key roles in regulating iridophore identity.

### Conclusion

To contribute to the understanding of pigment cell biology, we have developed a method for rapidly and reliably purifying melanocytes, iridophores, and retinal pigmented epithelium from zebrafish embryos, followed by global gene expression analysis by mRNA sequencing. This work represents the first concomitant comparison of three pigment cell types and whole zebrafish embryos, which uniquely allowed us to identify co-expressed genes indicative of shared function or developmental origin, as well as those that are specifically enriched in single cell types. We thus have identified many genes previously not reported to be enriched in these pigment cell types. In particular, we discovered a dramatic upregulation of specific enzymes from several metabolic pathways that coordinate guanine synthesis in iridophores, along with many membrane-trafficking components and transcription factors that are likely critical for iridophore identity. This characterization of global gene expression data from multiple purified zebrafish pigment cell types will provide a resource for further biological analysis of these cells.

## Supporting Information

Figure S1 **RPE Correlations.** Shown in (A) are the RPKMs of RPE_5_143hpf_32, which was collected at 143hpf, compared to the average RPKMs of RPE libraries 1–4, which were collected at 77–86hpf, for the 9029 genes detected at an average of 1–5000 RPKM across all five RPE libraries. Shown in (B) are the RPKM values for all genes detected between 1 and 5000 RPKM by RPE samples held at 25°C and 32°C. Magenta circles represent the 24 genes described as RPE-enriched in our analysis compared to iridophores, melanocytes, and whole embryos (r = 0.95).
(TIF)

Figure S2 **Melanocyte Time Point Correlations.** Shown are scatterplots of RPKM values for all genes expressed between 1 and 5000 RPKM by melanocytes collected at different time points.
(TIF)

Figure S3 **Technical Replicate Correlation.** Shown are scatterplots of RPKM values obtained for the technical sequencing replicates of sample Mel_3_77hpf. Sequencing runs 351 and 433 were single end reads of 36 and 42 nucleotides, respectively. 399s61 and 399s62 represent the two ends of a paired end 101 sequencing run.
(TIF)

Figure S4 **Quantitative RT-PCR and mRNA sequencing expression data are correlated.** Examined genes are indicated by object shape, as in the legend on the lower right. Cell type comparisons are indicated by color in the legend on upper left. The purple line indicates the position of perfect correlation. RNA-seq fold changes are computed directly for each cell type comparison [i.e. log2(melanocyte RPKM/iridophore RPKM) ]. QPCR fold changes are calculated by first normalizing expression relative to beta actin, followed by the log transformation.
(TIF)

Figure S5 **Transcriptome Coverage.** The number of genes identified (y-axis) per number of sequence reads (x-axis) obtained is plotted for each sample used in this analysis. Technical replicates are shown as individual lines, colored by library type as indicated on the lower right. For example, the three green lines represent the three technical sequencing replicates of the pooled 3dpf whole embryos cDNA library. The dashed vertical line is at one million reads.
(TIF)

Figure S6 **Pearson correlations of RNA-seq expression data.** Genes are ordered by increasing whole embryo expression (Y-axis). Each point represents the correlation value for the 1000 gene-window between the indicated cell-type comparison, beginning at that position. The most highly expressed genes in whole embryos are ribosomal proteins, which correspond to a slight peak in correlation values when compared to melanocytes (lower right). The average Pearson correlations across all windows for each comparison are indicated on the right, with corresponding horizontal lines.
(PDF)

Figure S7 **Whole embryos RNA-seq fold change bias.** QPCR-based fold change values demonstrate a systematic overcalling of fold change values upon comparison of randomly fragmented whole embryo cDNA libraries with reduced-representation pigment cell libraries. The purple line represents the position of perfect correlation.
(TIF)

Table S1 **Sequencing Summary.** Gross sequencing results for cDNA libraries used in this analysis, with their respective developmental time points at 28.5C. Unless indicated, fish used for each library were held at 25°C. The type of sequencing runs are indicated by the suffixes of technical replicate names: 351: single end 36, 433, 436, and 440: single end 42, 399s61 and 399s62: paired end 101. *Uniquely mapped to the Washington University non-redundant cDNA database. Tags that were not uniquely assigned to a gene typically mapped to polyA, genomic, and Illumina-adapter sequences. **Total RNA was used for Illumina library preparation of 20 pooled 3dpf embryos, resulting in a lower fraction of uniquely mapping sequences due to a large proportion of ribosomal RNA in library. Abbreviations: Mel - Melanocyte, Irid - Iridophore, RPE - Retinal Pigmented Epithelium, hpf - hours post fertilization, dpf - days post fertilization.
(XLSX)

Table S2 **All Libraries RPKM.** Normalized expression values (RPKM) for each of the libraries used in this analysis.
(XLS)

Table S3 **Primers.** Primers used for library preparation and qPCR analysis.
(XLSX)

Table S4 **Non-redundant cDNA database.** Sequences for the genes used in this analysis.
(XLSX)

Table S5 **Pigment Cell Expression AvgRPKM pValues.** Averaged RPKM expression values for melanocyte, RPE, iridophore, and whole embryo. Also shown are Student’s T-test p-values, ensembl transcript and gene reference numbers, and the corresponding zv9 genomic location of each gene.
(XLSX)

Table S6 **Melanocyte & RPE Shared. Genes expressed by both melanocytes and RPE with an RPKM 2-fold greater than iridophores, and 8-fold greater than whole embryos.**
(XLSX)

Table S7 **Melanocyte & Iridophore Shared.** Genes expressed by both melanocytes and iridophores with an RPKM 2-fold greater than RPE, and 8-fold greater than whole embryos.
(XLSX)

Table S8 **RPE & Iridophore Shared.** A single gene is expressed by both RPE and iridophores with an RPKM 2-fold greater than melanocytes, and 8-fold greater than whole embryos
(XLSX)

Table S9 **Melanocyte Enriched Genes.** Genes expressed in melanocytes 2-fold greater than RPE and iridophores, and 8-fold greater than whole embryos.
(XLSX)

Table S10 **Iridophore Enriched Genes.** Genes expressed in iridophores 2-fold greater than RPE and melanocytes, and 8-fold greater than whole embryos.
(XLSX)

Table S11 **RPE Enriched Genes.** Genes expressed in RPE 2-fold greater than iridophores and melanocytes, and 8-fold greater than whole embryos.
(XLSX)
