# Supplementary figures and images for: Gene Expression Analysis of Zebrafish Melanocytes, Iridophores, and Retinal Pigmented Epithelium Reveals Indicators of Biological Function and Developmental Origin
Source: PLoS One. 2013 Jul 9;8(7):e67801. doi: 10.1371/journal.pone.0067801 (PMC3706446; doi:10.1371/journal.pone.0067801)

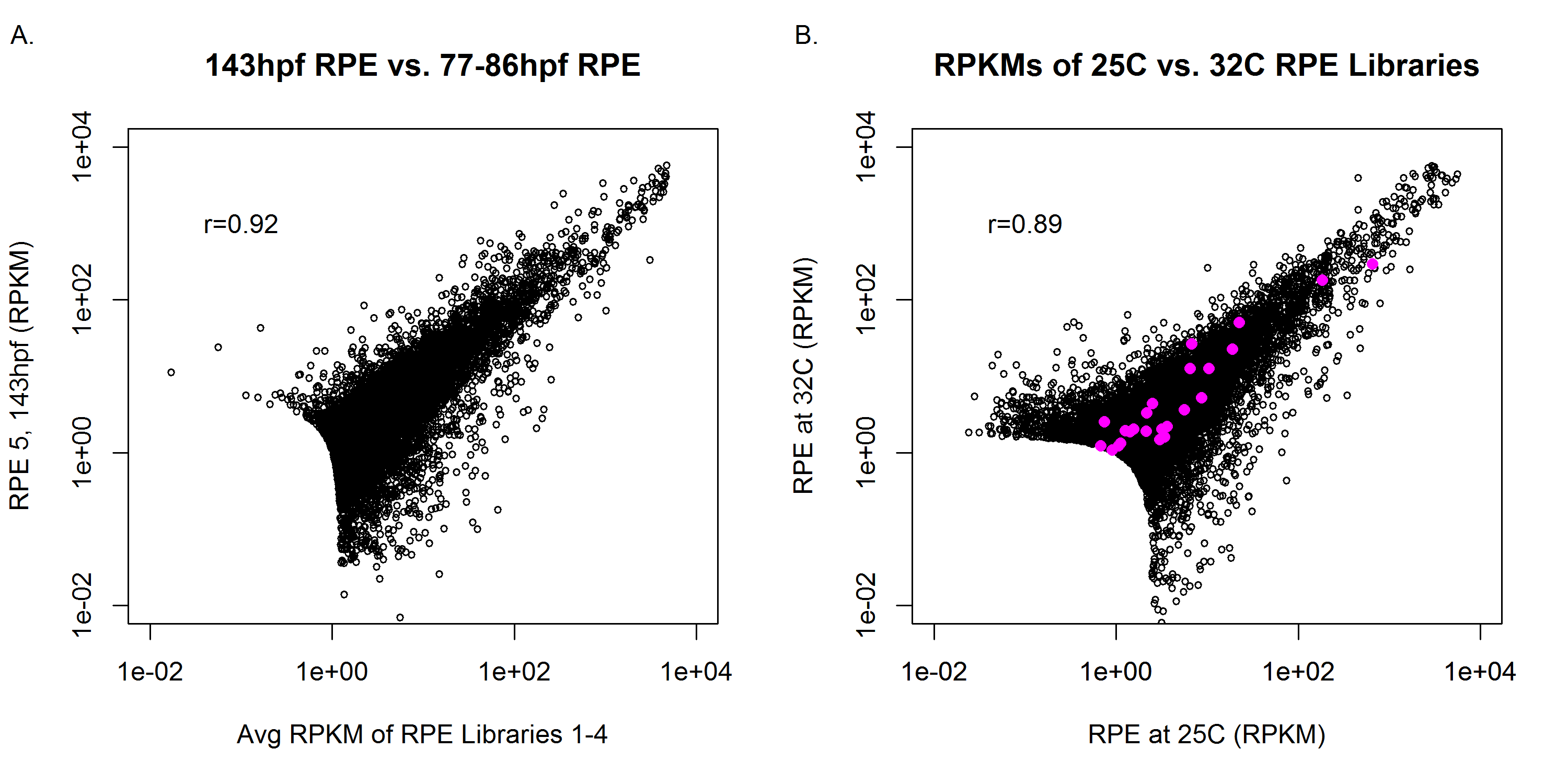

Supplement: Figure S1 — RPE Correlations. Shown in (A) are the RPKMs of RPE_5_143hpf_32, which was collected at 143hpf, compared to the average RPKMs of RPE libraries 1–4, which were collected at 77–86hpf, for the 9029 genes detected at an average of 1–5000 RPKM across all five RPE libraries. Shown in (B) are the RPKM values for all genes detected between 1 and 5000 RPKM by RPE samples held at 25°C and 32°C. Magenta circles represent the 24 genes described as RPE-enriched in our analysis compared to iridophores, melanocytes, and whole embryos (r = 0.95). (TIF) [file pone.0067801.s001.tif]

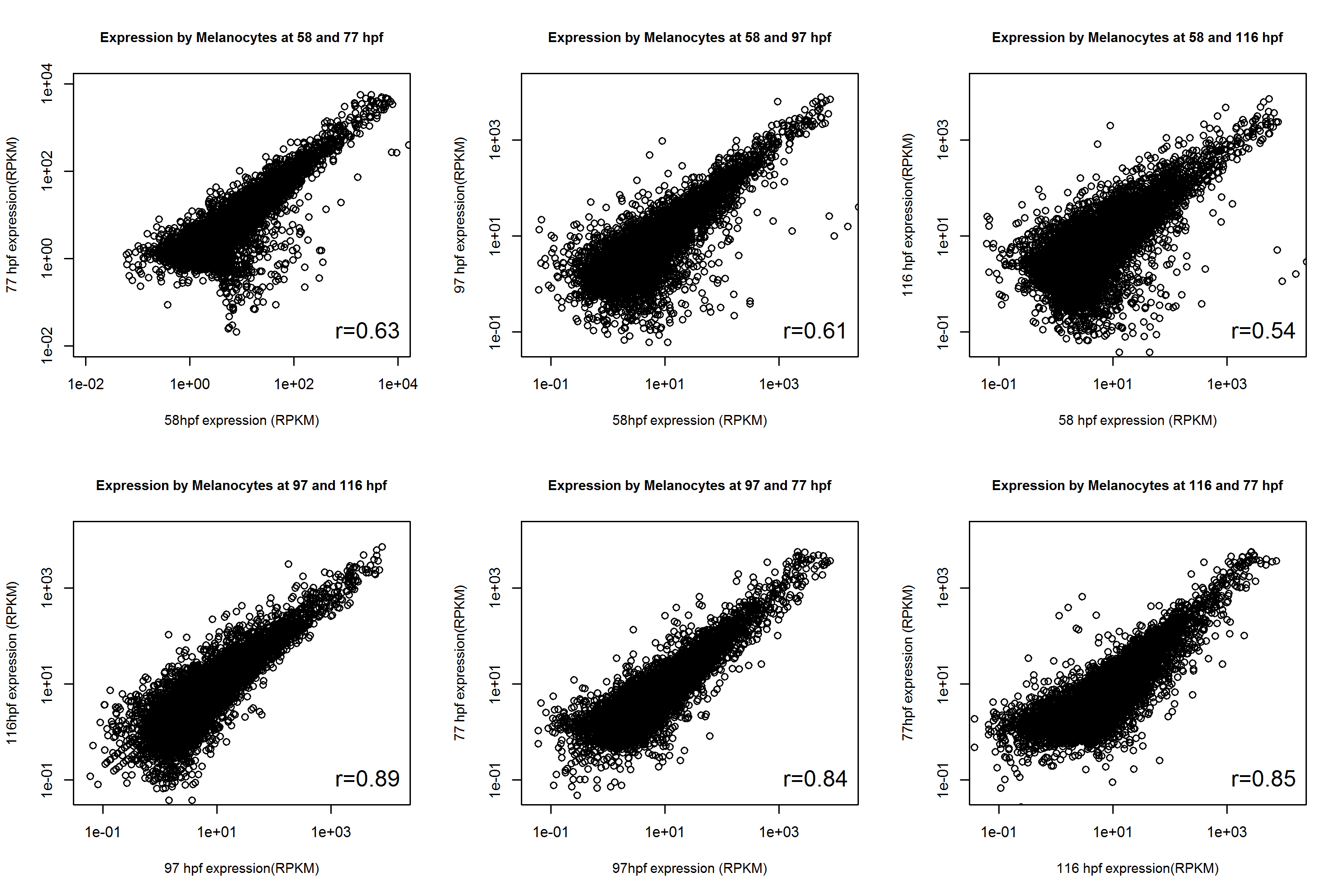

Supplement: Figure S2 — Melanocyte Time Point Correlations. Shown are scatterplots of RPKM values for all genes expressed between 1 and 5000 RPKM by melanocytes collected at different time points. (TIF) [file pone.0067801.s002.tif]

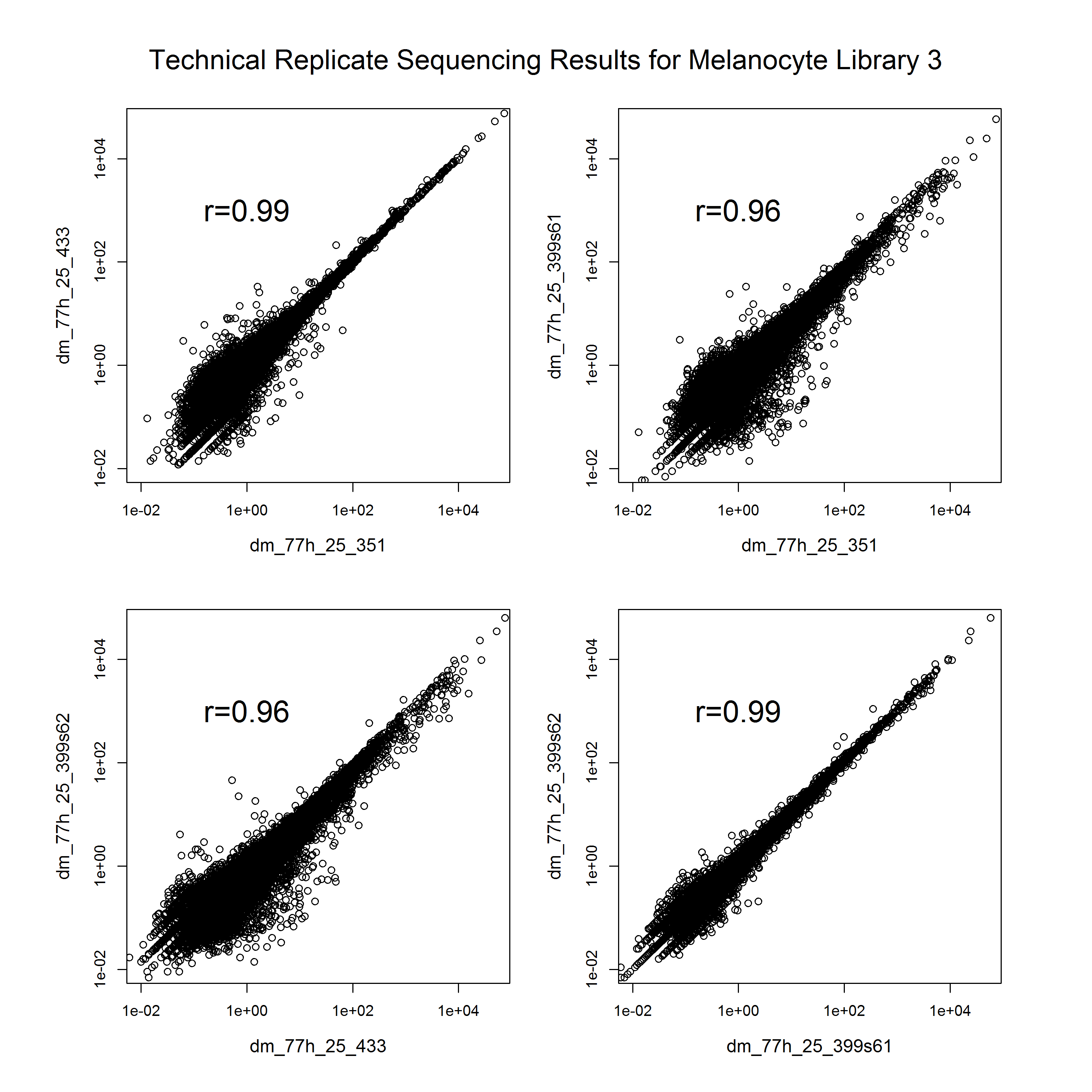

Supplement: Figure S3 — Technical Replicate Correlation. Shown are scatterplots of RPKM values obtained for the technical sequencing replicates of sample Mel_3_77hpf. Sequencing runs 351 and 433 were single end reads of 36 and 42 nucleotides, respectively. 399s61 and 399s62 represent the two ends of a paired end 101 sequencing run. (TIF) [file pone.0067801.s003.tif]

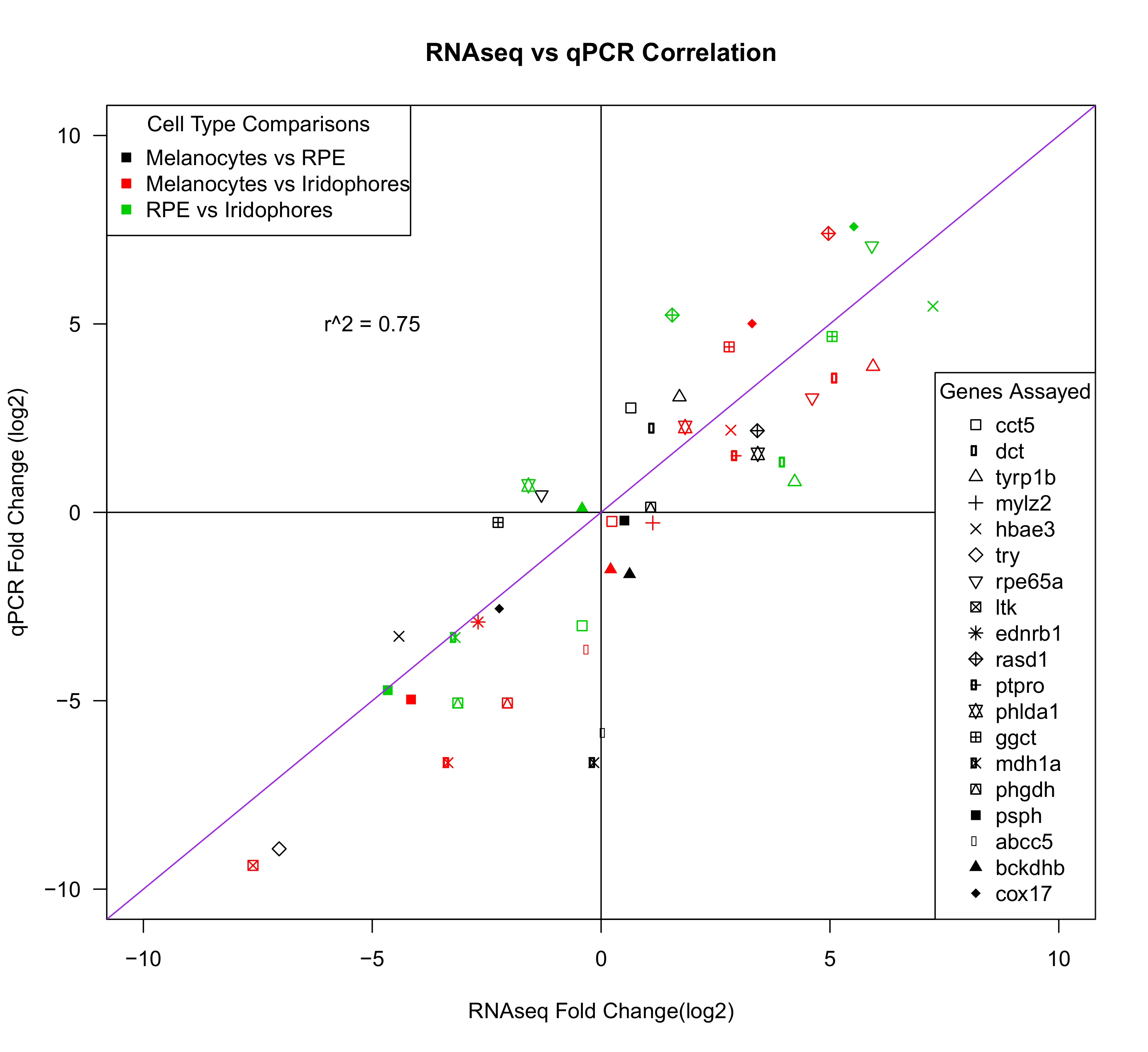

Supplement: Figure S4 — Quantitative RT-PCR and mRNA sequencing expression data are correlated. Examined genes are indicated by object shape, as in the legend on the lower right. Cell type comparisons are indicated by color in the legend on upper left. The purple line indicates the position of perfect correlation. RNA-seq fold changes are computed directly for each cell type comparison [i.e. log2(melanocyte RPKM/iridophore RPKM) ]. QPCR fold changes are calculated by first normalizing expression relative to beta actin, followed by the log transformation. (TIF) [file pone.0067801.s004.tif]

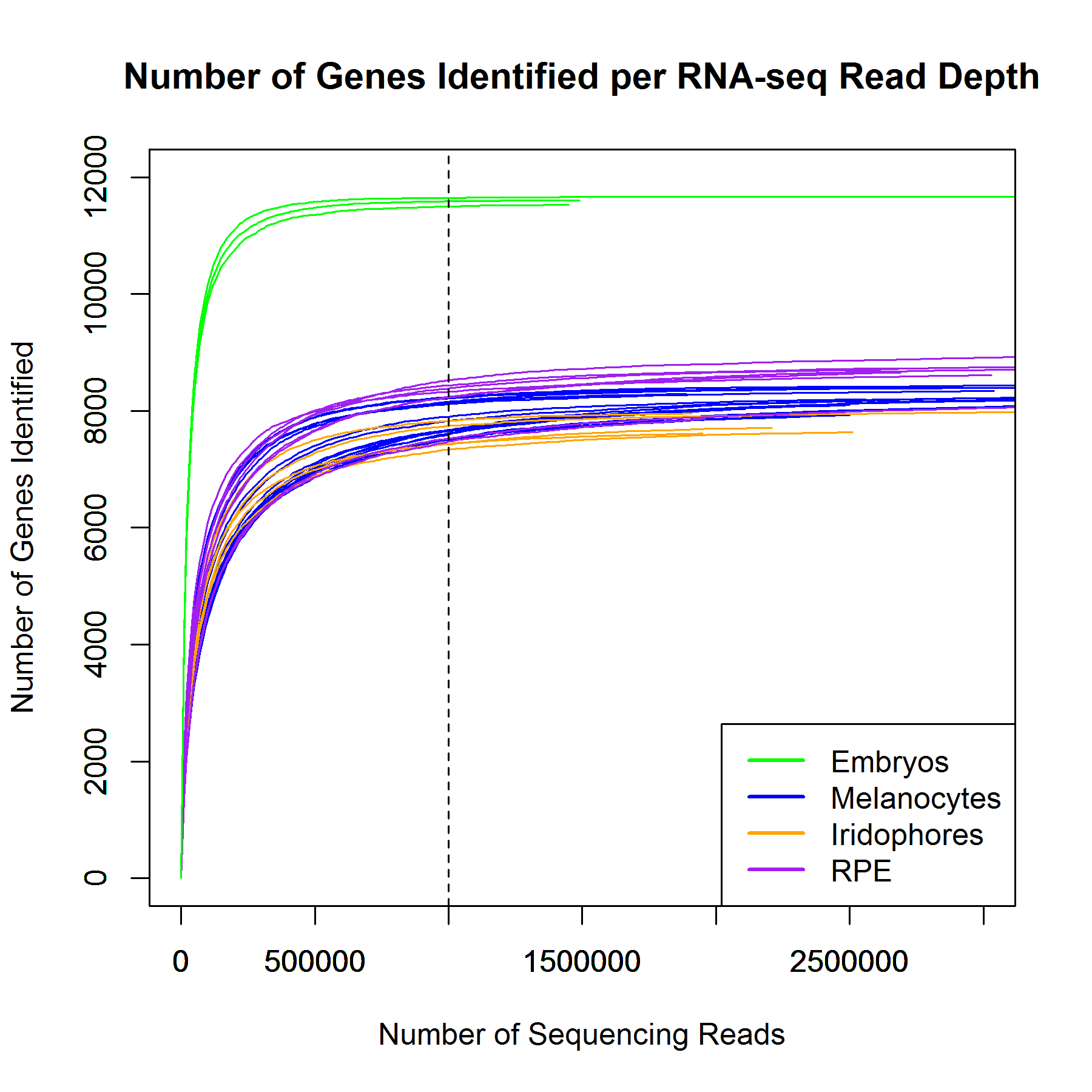

Supplement: Figure S5 — Transcriptome Coverage. The number of genes identified (y-axis) per number of sequence reads (x-axis) obtained is plotted for each sample used in this analysis. Technical replicates are shown as individual lines, colored by library type as indicated on the lower right. For example, the three green lines represent the three technical sequencing replicates of the pooled 3dpf whole embryos cDNA library. The dashed vertical line is at one million reads. (TIF) [file pone.0067801.s005.tif]

# Pearson Correlation of RNAseq Data

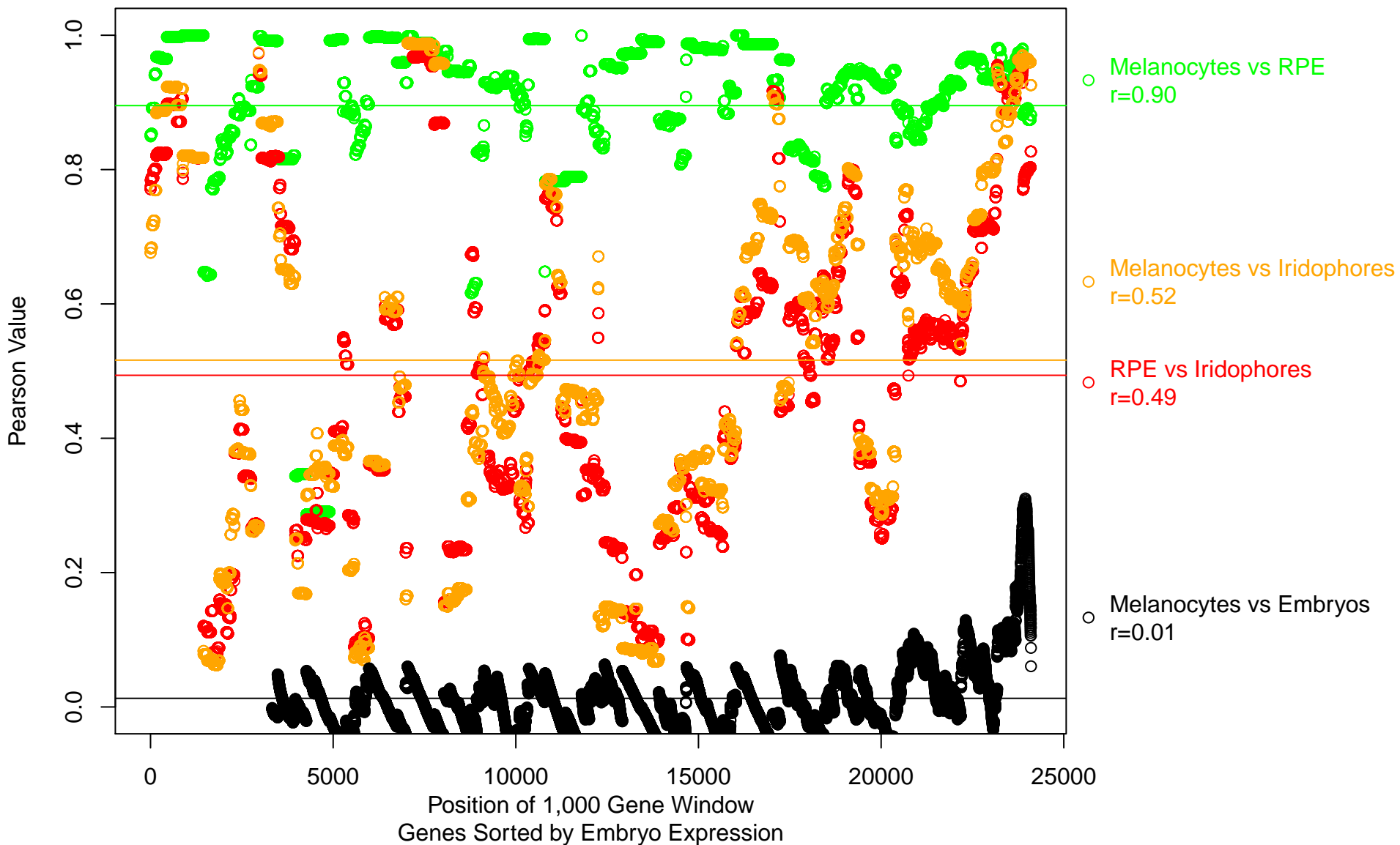

Supplement: Figure S6 — Pearson correlations of RNA-seq expression data. Genes are ordered by increasing whole embryo expression (Y-axis). Each point represents the correlation value for the 1000 gene-window between the indicated cell-type comparison, beginning at that position. The most highly expressed genes in whole embryos are ribosomal proteins, which correspond to a slight peak in correlation values when compared to melanocytes (lower right). The average Pearson correlations across all windows for each comparison are indicated on the right, with corresponding horizontal lines. (PDF) [file pone.0067801.s006.pdf]

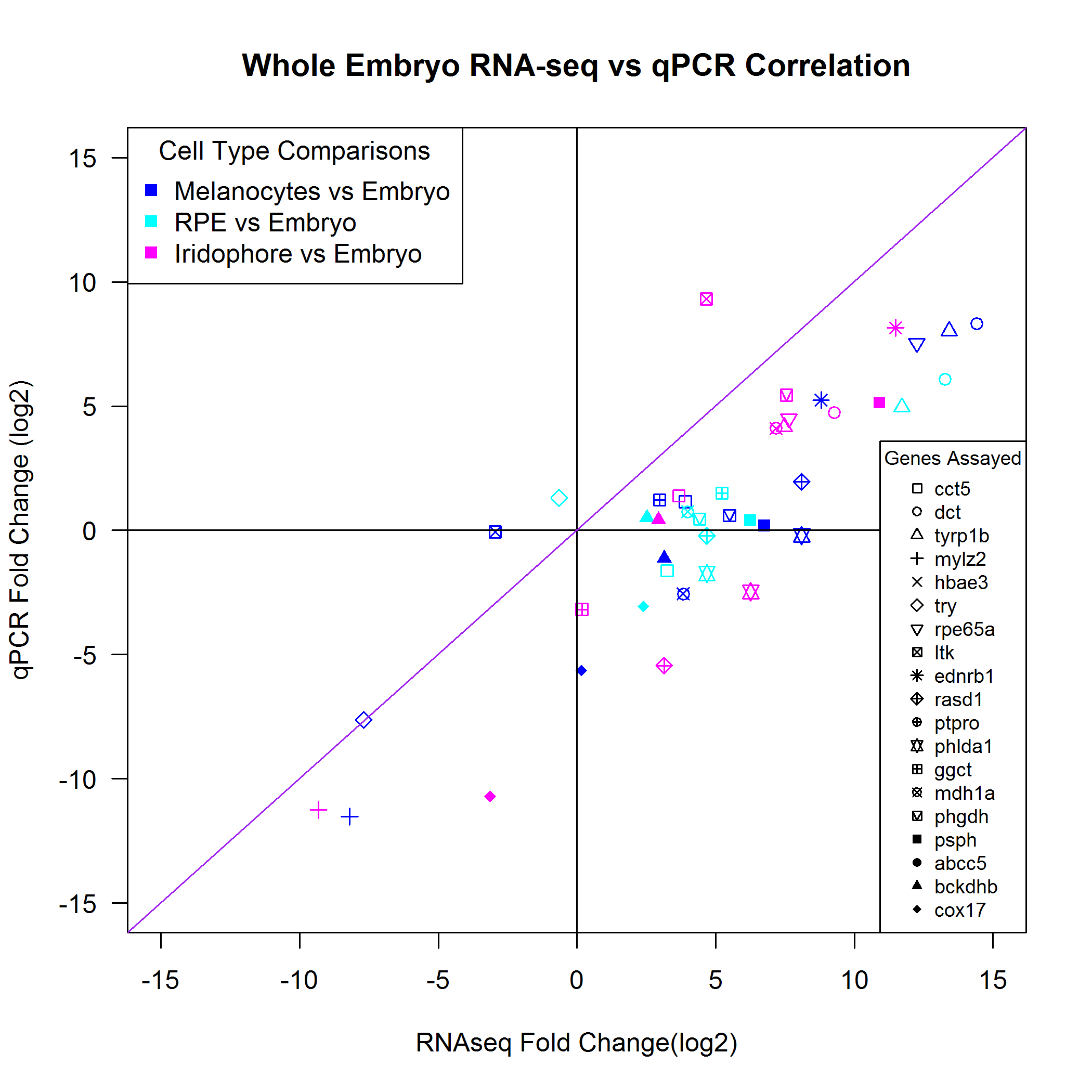

Supplement: Figure S7 — Whole embryos RNA-seq fold change bias. QPCR-based fold change values demonstrate a systematic overcalling of fold change values upon comparison of randomly fragmented whole embryo cDNA libraries with reduced-representation pigment cell libraries. The purple line represents the position of perfect correlation. (TIF) [file pone.0067801.s007.tif]
